# Applications of donor-derived cell-free DNA in kidney transplantation healthcare: view from a prospective single-center study

**DOI:** 10.1007/s10157-026-02889-8

**Published:** 2026-06-02

**Authors:** Phuong Thanh Nguyen, Hirofumi Nakaoka, Shigeki Mitsunaga, Hiromichi Aoyama, Hiroshi Kitamura, Kenichi Saigo, Ituro Inoue

**Affiliations:** 1 Department of Genetics, The Graduate University for Advanced Studies (SOKENDAI), Shizuoka, Japan; 2 Human Genetics Laboratory, National Institute of Genetics, Shizuoka, Japan; 3 Institute of Biology, Vietnam Academy of Science and Technology, Hanoi, Vietnam; 4 Department of Cancer Genome Research, Sasaki Institute, Sasaki Foundation, Tokyo, Japan; 5 Department of Biomedical Data Science, Kagoshima University Graduate School of Medical and Dental Sciences, Kagoshima, Japan; 6 Department of Pediatric Surgery, Keio University School of Medicine, Tokyo, Japan; 7 iSan Bio Inc, Yokohama, Japan; 8 Department of Surgery, National Hospital Organization Chiba-East Hospital, Chiba, Japan; 9 Department of Transplantation Surgery, Japan Community Healthcare Organization Chiba Hospital, Chiba, Japan; 10 Department of Pathology, National Hospital Organization Chiba-East Hospital, Chiba, Japan; 11 Toyo Medical Clinic Oami, MEISEIKAI MEDICAL Corporation, Chiba, Japan

**Keywords:** Donor-derived cell-free DNA, Kidney transplant, Liquid biopsy, Acute rejection, Immunosuppressant, Targeted sequencing

## Abstract

**Background:**

Non-invasive biomarkers are essential for monitoring transplant health, particularly for detecting acute rejection. Donor-derived cell-free DNA (dd-cfDNA) has emerged as a promising biomarker in solid organ transplantation, but optimized methodology and comprehensive evaluation in kidney transplantation (KTx), especially in Japan, are currently limited.

**Methods:**

We developed a customized approach for dd-cfDNA quantification and evaluated its utility as a biomarker in KTx. In this prospective longitudinal study, 322 blood samples were collected from 39 KTx recipients, mostly within the first year and up to 5 years post-transplant. Quantification was performed using a modified targeted sequencing protocol using a 1,000-SNP probe panel tailored to East Asian genetic backgrounds. The resulting dd-cfDNA values were correlated with clinical parameters and histological findings.

**Results:**

General longitudinal dynamics of dd-cfDNA in KTx patients were comprehensively described. Elevated dd-cfDNA was consistently associated with acute rejection and graft injuries. In particular, dd-cfDNA significantly distinguished rejection from non-rejection episodes (*p* = 1.9 × 10^− 4^) and showed moderate consistency with serum markers and biopsy findings. Noticeable surges of dd-cfDNA were reported in antibody-mediated rejection and various forms of kidney injury. Distinct from conventional serum markers, dd-cfDNA exhibited a significant inverse correlation with immunosuppressant trough levels (Spearman’s rho = − 0.25, *p = *8 × 10^− 3^).

**Conclusions:**

Our study demonstrated that dd-cfDNA is a robust, non-invasive biomarker for detecting allograft rejection and injury in KTx and is superior for monitoring patients’ immunosuppressive response. We also present a scalable, cost-effective sequencing-based methodology for quantifying dd-cfDNA, suitable for clinical application.

**Supplementary Information:**

The online version contains supplementary material available at 10.1007/s10157-026-02889-8.

## Introduction

Kidney transplantation (KTx) is often the last resort for patients with end-stage kidney diseases, offering benefits that can significantly enhance a patient’s quality of life [1]. However, renal acute rejection (AR), affecting 5.4% to 8.8% of adult KTx recipients within the first-year post-transplant (post-Tx) [2, 3], poses a substantial risk for graft failure [4]. AR is characterized by interstitial inflammation and tubulitis, frequently accompanied by microvascular injury such as glomerulitis, peritubular capillaritis, or arteritis, as defined by the Banff classification [5].

Advancements in pre- and post-transplant care for KTx, such as improved HLA matching, preemptive interventions, and effective immunosuppressant therapies, have greatly reduced the incidence of AR in KTx [3, 6, 7]. Assessment of allograft kidney typically involves monitoring serum markers such as creatinine, cystatin C, blood urea nitrogen and serum procalcitonin, as well as estimated glomerular filtration rate (eGFR) or proteinuria [6, 8]. In cases of significant clinical concern, renal biopsy remains the gold standard for diagnosing acute rejection [5, 9], however, its invasiveness and associated risks restrict use to for-cause indications rather than routine protocol surveillance [10, 11].

Donor-derived cell-free DNA (dd-cfDNA), which is released from disrupted cells of transplanted organ, can be detected in the recipient’s blood plasma or urine, where its amount and variability can inform the status of graft integrity [12, 13]. A growing number of studies have focused on quantifying dd-cfDNA to enhance the diagnosis of AR and other forms of allograft injury [14–23]. However, long-term observation using a scalable methodology for monitoring dd-cfDNA in Japanese KTx is still lacking.

Here, utilizing a SNP panel designed specifically for the East-Asian population, we examined longitudinal dd-cfDNA levels in KTx patients from a single-center cohort for up to five years post-Tx. Elevated dd-cfDNA levels provided early indications of AR episodes and diverse forms of graft injury. The relation of dd-cfDNA was observed not only with clinical parameters and biopsy-derived histological assessments but also with immunosuppressant dosages. Our study validated the potential of dd-cfDNA as a non-invasive biomarker for evaluating graft integrity in KTx. Furthermore, we established a proof of concept using a cost-effective targeted sequencing protocol to quantify dd-cfDNA, highlighting its applicability in clinical settings.

## Materials and methods

### Cohort description

This single-center prospective study included 39 kidney transplant donor–recipient pairs recruited from the National Hospital Organization Chiba-East Hospital (Chiba, Japan) with written consent. Living donors were predominantly related to recipients, most commonly spouses (43.6%), followed by parent–child pairs (23.1%) and siblings (17.9%). The primary renal diagnoses pre-operation were IgA nephropathy and diabetic nephropathy (30.8% and 20.5% of all cases, respectively). Patients with markedly high preoperative donor-specific antibodies (DSA) levels were not included in this study. Donor–recipient relationships and corresponding number of informative SNPs (see Supplementary Methods) are summarized in Fig. S1. The study protocol was approved by the institutional ethics committee and was conducted in accordance with the Declaration of Helsinki and in compliance with the principles of the Declaration of Istanbul. Demographic summary of KTx cases is presented in Table 1.

**Table 1:** Demography of kidney transplant cases and samples summary

Total number of Tx recipients	39
**Recipient characteristics**	*n* (%)
Gender (male/female)	25 (64.1%) / 14 (35.9%)
Age, median (range)	48 (24–69)
Longitudinal follow-up cases	36 (92.3%)
Cross-sectional cases	3 (7.7%)
**Donor-recipient relationship**	*n* (%)
Spouse	17 (43.6%)
Parent-to-child	9 (23.1%)
Sibling	7 (18.0%)
Others (relatives, in-law)	6 (15.4%)
**Total number of plasma sample**	*n *= 322
Sample collected per patient, median (IQR)	9 (3)
Collected within 10 days post-transplantation, *n* (%)	143 (44.5%)
Collected after 10 days, *n* (%)	178 (55.5%)
Total number of samples collected with biopsy, *n* (%)	66 (20.6%)
Long-term longitudinal samples (> 3 years), *n* (%)	21 (6.5%)
Long-term cross-sectional samples (> 3 years), *n* (%)	10 (3.1%)
**Sample collection day in regard to biopsy day**	*n*
0 (Day of biopsy)	36
within 1 day	11
within 2 days	3
within 3–6 days	15

## Sample collection and clinical assessments

After genotyping both KTx donors and recipients, we collected a total of 322 blood samples from the recipients (i.e., KTx patients) at multiple time points, ranging from the first day to 5 years post-transplant. Specifically, one-third of the patients had samples obtained within the first 28 days post-Tx, while half of the patients (*n* = 19) had samples collected up to the 1-year time point. Samples were collected spanning the period from 2016 to 2021.

Regarding sampling type, majority of samples were collected at fixed timepoints (i.e., protocol sampling, *n* = 241), including within the first several weeks, at one month, and at one year post-Tx. A portion of samples were collected on unprompted occasions with clinical concerns (i.e., for-cause sampling, *n* = 37), especially where AR is suspected. Criteria for for-cause sampling included a ≥ 20% increase in serum creatinine from baseline, new or worsening proteinuria (> 0.5 g/day), or other unexplained deterioration in graft function. We also measured dd-cfDNA levels of samples collected 3 to 5 years post-Tx (regarded as long-term sampling, *n* = 21) (Table 1). The remaining samples (*n* = 22) were obtained to evaluate the impact of biopsy on dd-cfDNA levels.

Renal biopsy was performed with ultrasound-guided needle biopsy and histopathological examination was assessed and classified according to the Banff classification current at the time of evaluation by renal pathologist. HLA antibody screening (FlowPRA) was performed for pre-Tx and post-Tx specimens, especially for cases with suspected rejection. Positive screening tests were followed up with single antigen test (WAKFlow HR) to identify the presence of donor specific antibodies. Intra-graft C4d stain was performed to verify active antibody–mediated rejection (ABMR). Following the biopsy-based Banff classification guidelines [5, 9], AR was identified in six patients, including two cross-sectional cases enrolled upon hospitalization long after kidney transplantation (i.e., Patient #23 and #24). In total of the nine reported AR episodes, two were ABMR, and seven were acute T cell-mediated rejection (TCMR). All clinical characteristics are summarized in Table 2 (see Supplemental Material 1 for detail).

**Table 2:** Summary of clinical characteristics of kidney transplant patients

Transplant prescreening	*n* (%)
Preemptive transplant	15 (38.5%)
ABO Incompatible	12 (30.8%)
**Primary renal diagnoses** (include suspected cases)	*n* (%)
IgA nephropathy	11 (28.2%)
Diabetic nephropathy	8 (20.5%)
Chronic glomerulonephritis	4 (10.3%)
Chronic nephritis	3 (7.7%)
Congenital anomalies (renal hypoplasia, hydronephrosis)	3 (7.7%)
Nephrosclerosis	3 (7.7%)
Unknown etiology	3 (7.7%)
Other (include Systemic lupus erythematosus, Membranoproliferative glomerulonephritis, Alport syndrome and Polycystic kidney disease)	4 (12.8%)
**Transplant operative parameters**	Mean (± SD)
Donor operation time (minute)	215.1 (± 50.7)
Donor bleeding amount (mL)	45.3 (± 111.7)
Recipient Operation time (minute)	370.2 (± 76.8)
Recipient bleeding amount (mL)	226.0 (± 164)
Warm ischemic time (second)	324.7 (± 153.6)
Total ischemic time (minute)	108.2 (± 51.3)
**Clinical renal markers**	Median (IQR)
Serum creatinine (mg/dL)	1.95 (1.75)
Cystatin C (mg/L)	1.91 (0.88)
Blood urea nitrogen (mg/dL)	25.8 (16.2)
**Immunosuppression treatments**	Number of patient (*n*)
Tacrolimus (FK506)	*n* = 35
Mycophenolate mofetil (MMF)	*n* = 39
Cyclosporin A (CsA)	*n* = 4
Everolimus (EVR)	*n* = 6
Azathioprine (AZP)	*n* = 1
**Biopsy confirmed rejection**	Episode (number of patient)
T cell-mediated rejection (TCMR)	7 (*n* = 6)
Antibody-mediated rejection (ABMR)	2 (*n* = 2)

Procedures of cfDNA extraction, pooled-capture sequencing, dd-cfDNA quantification, and statistical analyses are described in the Supplementary Methods (in Supplementary Material 4).

## Results

### General dynamics of dd-cfDNA levels post-transplant

**Fig. 1:**
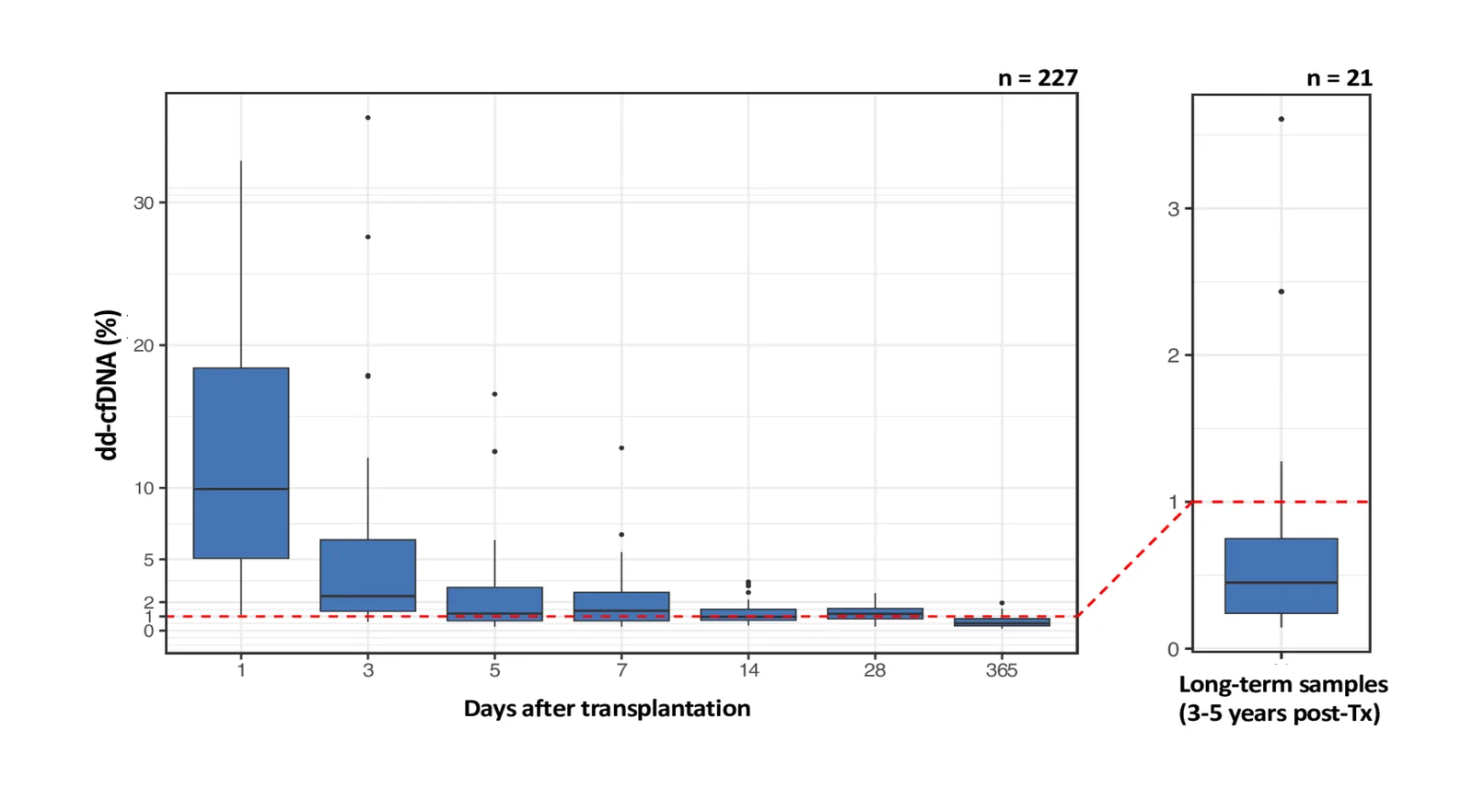
Overview of dd-cfDNA dynamic by protocol sampling within 1 year and after long period post-Tx (long-term samples); n = number of collected samples

The longitudinal dynamics of dd-cfDNA in 37 KTx patients with follow-up from day 1 to 1 year post-Tx are shown in Fig. 1. Significantly elevated dd-cfDNA levels were observed immediately after transplantation, reaching up to 30% and varying widely among patients. Consistent with previous studies, we observed a rapid exponential decline in dd-cfDNA level during the first week post-Tx, with the median reaching the 1% baseline by day 5. By day 14, 15 of 35 patients stabilized below 1%. At 1 year, most patients showed well-controlled graft status, with 14 of 19 maintaining dd-cfDNA levels < 1%. We additionally examined long-term follow-up patients (3–5 years post-Tx, *n* = 21), which revealed dd-cfDNA values typically below 1% (Fig. 1, right panel). This indicated the ongoing turnover of donor cells even in long-term stabilized grafts. Interestingly, only two long-term samples showed elevated dd-cfDNA levels: 3.61% in patient #8 at 5 years post-Tx and 2.43% in patient #31 at 3 years post-Tx, likely due to the reduced administration of immunosuppressant, such as tacrolimus (FK506).

**Fig. 2:**
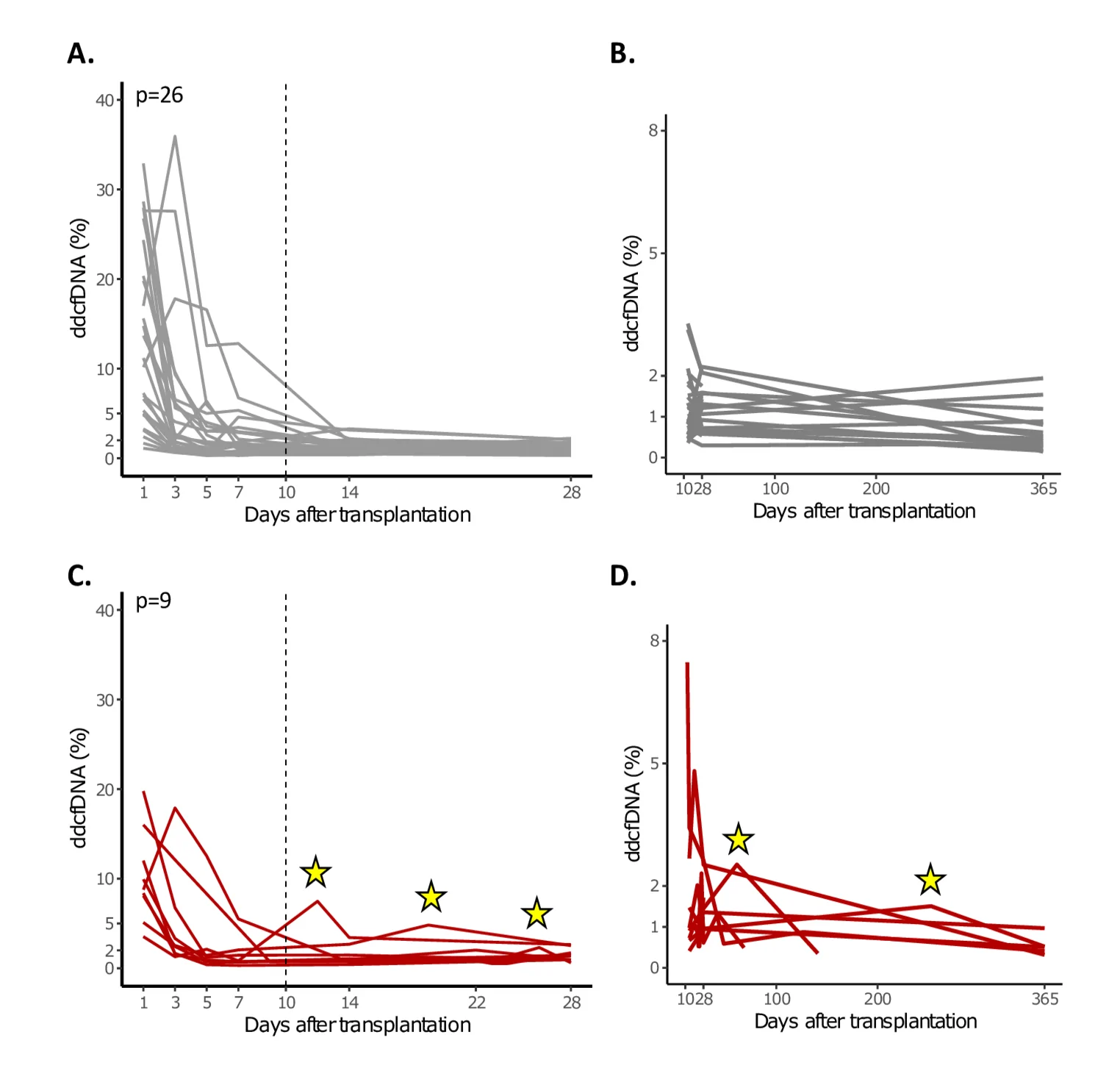
The dynamic of dd-cfDNA level among different groups of KTx patients. **a** The dynamic of dd-cfDNA level in the group of patients without any suspected episode of rejection (i.e., ‘eventless’ patients), within the first month post-Tx; **b** Follow-up on ‘eventless’ patients from day 10 to 1-year post-Tx; **c** The dynamic of dd-cfDNA level in the group of patients with suspected episodes of rejection (i.e., ‘event’ patient), within the first month post-Tx; **d** Follow-up on ‘event’ patients from day 10 to 1-year post-Tx. (Biopsy-confirmed rejection episodes are annotated with star shape; p = number of KTx patients)

From day 10 post-Tx onward, patients without suspected AR exhibited a ‘quiescent’ dd-cfDNA profile, with levels remaining consistently low throughout the first year post-Tx (Fig. 2a and b). In contrast, in the group of patients experiencing at least one event of suspected rejection (*n* = 9), dd-cfDNA levels fluctuated erratically throughout the period (Fig. 2c and d). Indeed, these peaks aligned with AR episodes confirmed by same-day histological assessments, validating the well-established link between dd-cfDNA surges and rejection events [19, 24, 25].

## dd-cfDNA as a sensitive indicator of rejection and other forms of kidney injuries

**Fig. 3:**
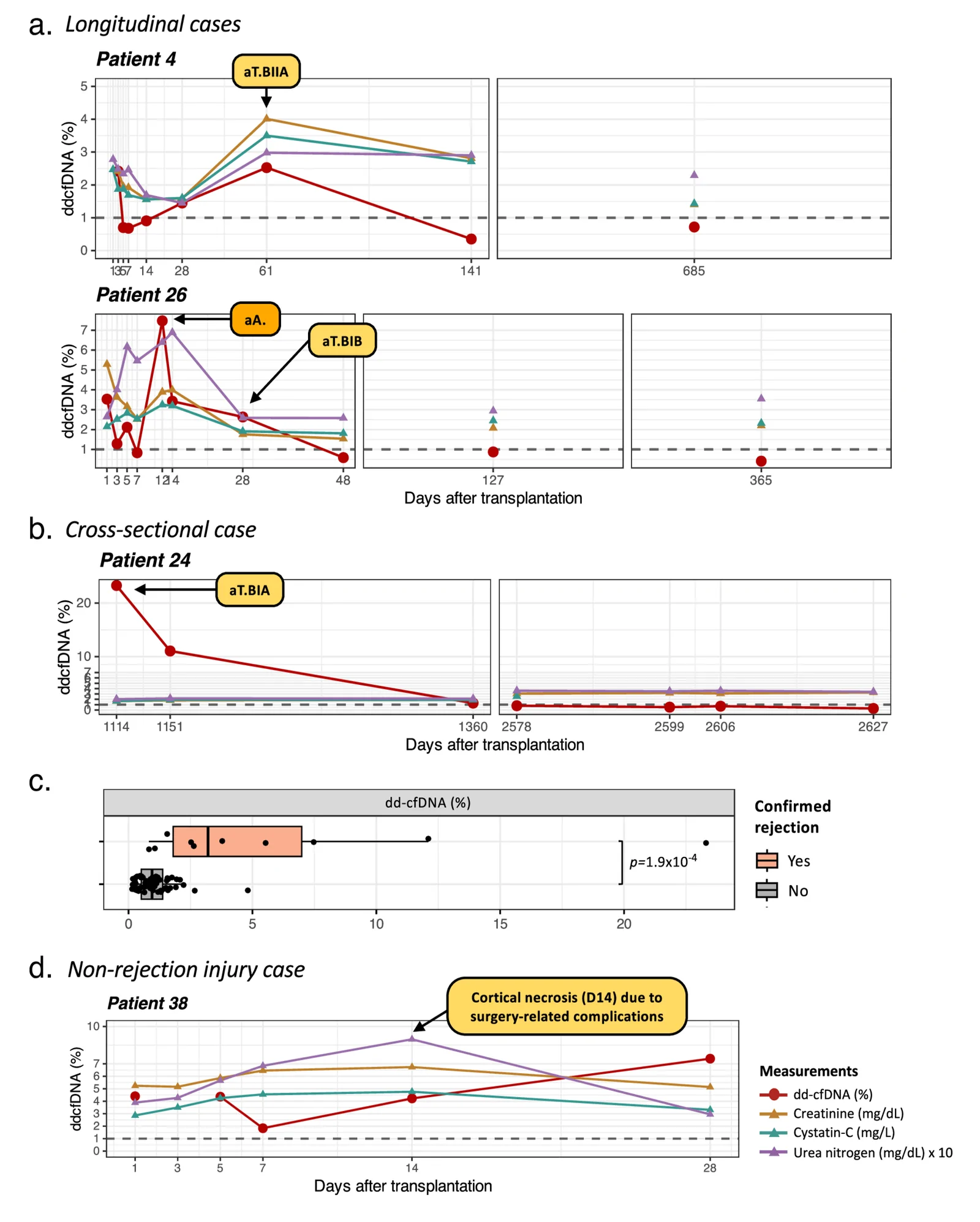
Longitudinal clinical measurements in representative cases of biopsy-confirmed rejection and other types of KTx injury. **a** Representative longitudinal cases with biopsy-confirmed rejections; **b** Representative cross-sectional case with biopsy-confirmed rejection upon hospitalization. Abbreviations: aT.BIA = acute T cell–mediated rejection Banff IA; aT.BIIA = acute T cell–mediated rejection Banff IIA; aT.BIB = acute T cell–mediated rejection Banff IB; aA. = active antibody–mediated rejection. **c** Comparison of dd-cfDNA levels between groups of biopsy-verified samples with and without rejection, Wilcoxon rank-sum test was performed; **d** Representative case of elevated dd-cfDNA level due to severe kidney injury not related to rejection

Case-by-case analysis showed that dd-cfDNA levels exceeded the 1% baseline in 8 of 9 samples from six patients with biopsy-confirmed AR (Fig.S2). Of these, longitudinal profiles revealed distinctive dd-cfDNA spikes at the time of biopsy-confirmed rejection (Fig. 3a). Notably, in two ABMR-confirmed samples, dd-cfDNA surged sharply, exceeding 7% in patient #26 and reaching up to 3.8% in patient #2. By comparison, TCMR was also associated with an increase in dd-cfDNA levels, albeit with delayed peaks and lesser extent, exemplified in patient #36 (Fig.S2A). Notably, in patient #2, an episode of active ABMR (sample 2R-E6; dd-cfDNA 3.78%) was associated with a marked increase in FlowPRA anti-HLA Class II reactivity, reaching 57.67% one month before biopsy and remaining elevated at 43.84% three months post-biopsy; subsequent single antigen test confirmed the presence of DSA. This observation exemplifies the responsiveness of dd-cfDNA to subclinical immunological activity associated with ABMR.

The two cross-sectional cases with ABMR (Patient #23 and #24) also exhibited extremely high levels of initial dd-cfDNA levels (Fig.S2B), with patient #24 exceeding 20% at hospitalization (Fig. 3b). Histological evidence confirmed significantly higher dd-cfDNA levels in AR versus non-rejection samples (*p* = 1.9 × 10^− 4^, Wilcoxon rank-sum test) (Fig. 3c). Other kidney function markers, including creatinine, cystatin C and blood urea nitrogen, also showed significant differences between groups (Fig.S3).

Interestingly, we also observed particularly high levels of dd-cfDNA in KTx patients with kidney injury not related to rejection, as in patient #15, diagnosed with severe calcineurin inhibitors toxicity (Fig.S2C), and in patient #38, diagnosed with cortical necrosis due to post-surgery complications (Fig. 3d). For the latter case, dd-cfDNA levels rose exceptionally high (over 7% at day 28 post-Tx), despite the lowered levels of other serum markers. This result emphasizes that although dd-cfDNA lacks the specificity to differentiate rejection from other causes of allograft injury (e.g., calcineurin inhibitor toxicity, infection, or acute tubular injury), its high sensitivity makes it a superior, broad biomarker for monitoring overall allograft integrity compared with conventional serum markers. It is worth noting that plasma dd-cfDNA may fluctuate with organ damage or invasive procedures, but our preliminary analysis showed that needle biopsy had no such effect (Fig.S4).

### The relationship between dd-cfDNA and conventional assessments of kidney integrity

**Fig. 4:**
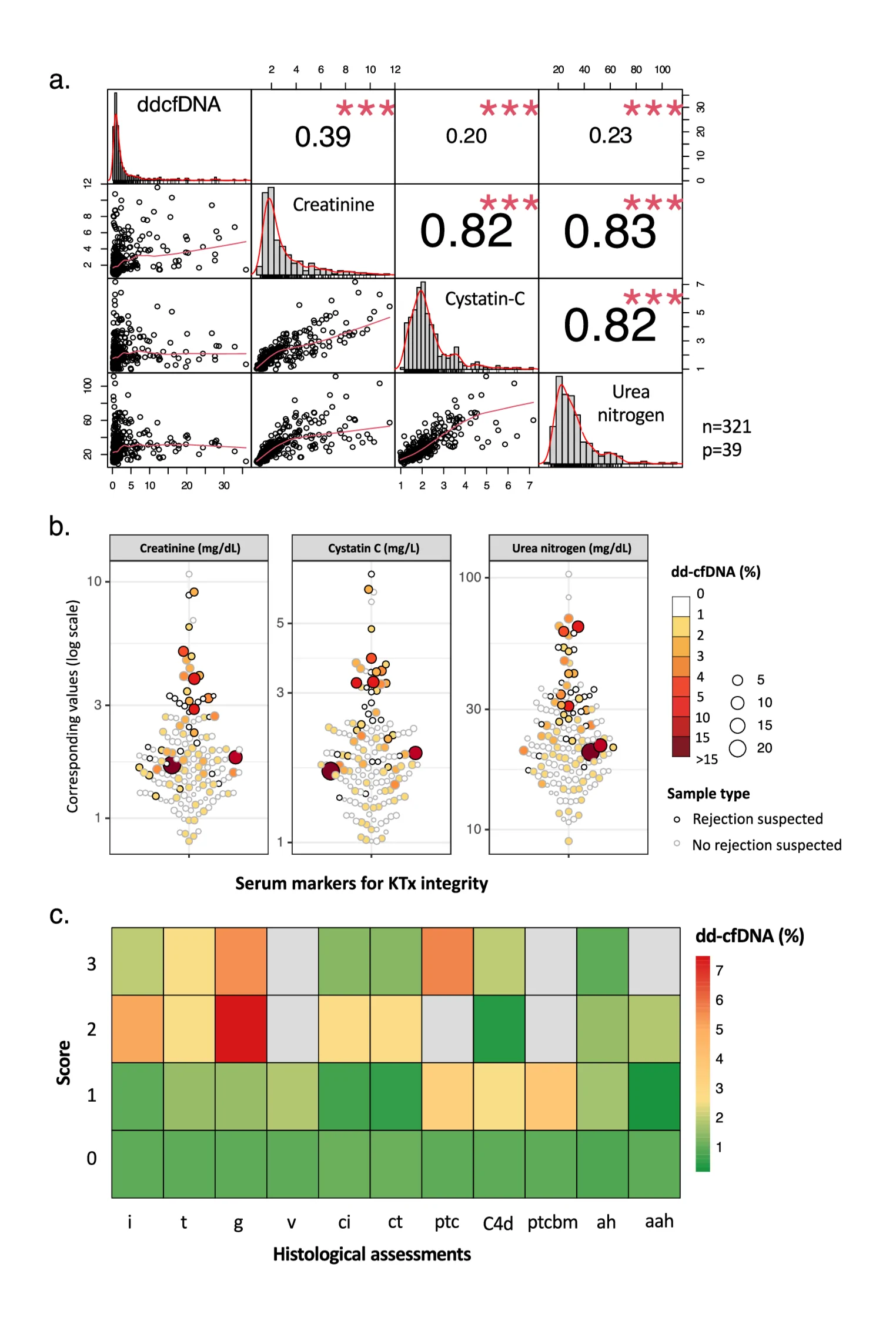
The relationship between dd-cfDNA and conventional assessments of kidney integrity. **a** Pairwise correlations between dd-cfDNA level and other serum markers. Spearman correlation was performed, n = number of samples, p = number of KTx patients; **b** Visualization of dd-cfDNA levels in relate to the distribution of serum markers for KTx integrity. Only samples taken at least 10 days post-Tx were plotted; dd-cfDNA levels are represented by both color intensity and dot size. **c** Integrative visualization of median dd-cfDNA level corresponding to various Banff Lesion Scores of histological assessments from biopsies (for *simplified visualization*, sub-scores are merged into the main corresponding scores, e.g., 1b → 1; 2b → 2; 3b → 3; 2.5 → 2); Abbreviation of Banff Lesion Score categories: *i* = Interstitial inflammation, *t* = Tubulitis, *g* = Glomerulitis, *v* = Intimal arteritis, *ci* = Interstitial fibrosis, *ct* = Tubular atrophy, *ptc* = Peritubular capillaritis, *C4d* = C4d staining scores, *ptcbm* = peritubular capillary basement membrane, *ah* = Arteriolar hyalinosis, *aah* = Hyaline arteriolar thickening

Given that elevated levels of dd-cfDNA and other serum markers aid in identifying KTx patients with AR, we anticipated strong correlations between them. However, this was not entirely the case, the three kidney function–related serum markers (i.e., creatinine, cystatin C, and blood urea nitrogen) exhibited strong pairwise correlations (Spearman’s rho > 0.82). In contrast, the correlation coefficients between dd-cfDNA and any of these markers were relatively low, ranging from 0.23 to 0.39 (*p* < 1 × 10^− 3^) (Fig. 4a). Elevated dd-cfDNA was more aligned with moderate to extreme elevations of serum markers (Fig. 4b), suggesting that dd-cfDNA may offer a unique perspective on allograft integrity compared to others due to its distinct origin and wide fluctuation range. Indeed, correlations between dd-cfDNA and serum markers remained significant in patients without rejection (“eventless” group) but became non-significant in those with suspected rejection (“event” group) (Fig.S5), indicating that dd-cfDNA dynamics become increasingly erratic in the presence of allograft injuries.

Biopsies showed that elevated dd-cfDNA levels generally aligned with higher Banff grades across multiple categories (Fig. 4c). All AR episodes typically exhibited high dd-cfDNA, regardless of the heterogeneity in Banff Lesion Scores (Fig. S6A). In contrast, chronic lesions such as arteriosclerosis and interstitial fibrosis and tubular atrophy (IF/TA) showed no consistent relationship with dd-cfDNA. Notably, high dd-cfDNA in AR cases sometimes coincided with only mild to moderate chronic scores (Fig. S6B), highlighting the limitation of biopsy-based evidence in recognizing potential subclinical injuries. In fact, detection of elevated dd-cfDNA levels during early-to-intermediate chronic stages may reveal hidden allograft damages, suggesting that integrating dd-cfDNA with histology could enhance diagnostic accuracy and allow earlier intervention.

**Fig. 5:**
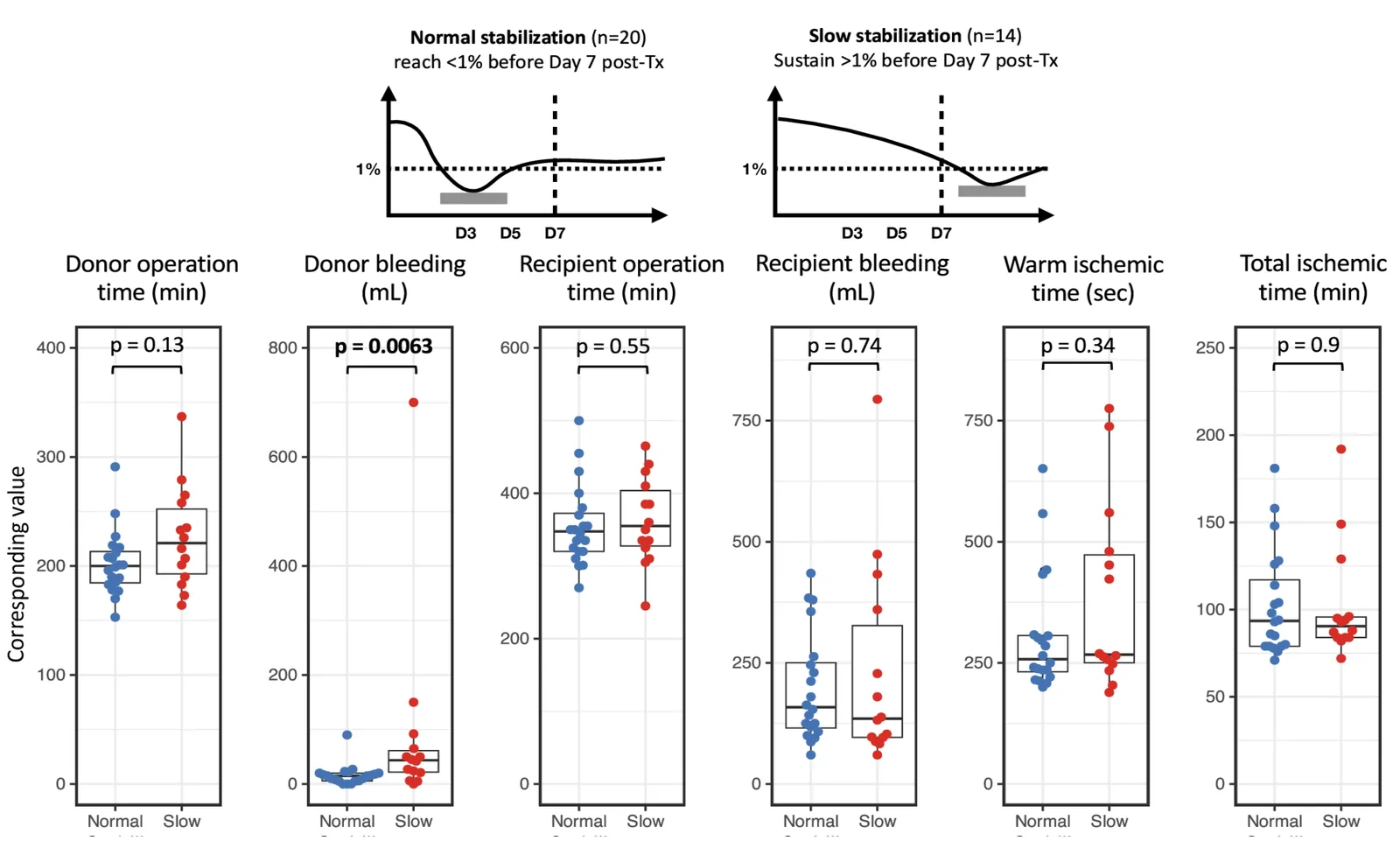
Comparison of surgical factors between groups of patients with typical (normal) stabilization curve of dd-cfDNA (dd-cfDNA reaches < 1% within 7 days post-Tx) versus those showing slow stabilization pattern (dd-cfDNA stays above 1% until 7 days post-Tx); Wilcoxon rank-sum tests were performed

We hypothesized that various surgical factors during KTx procedure might affect dd-cfDNA stabilization in recipients. By categorized KTx patients into two groups based on the patterns of dd-cfDNA dynamics within the first week post-Tx, the amount of donor bleeding differed significantly (*p* = 6.3 × 10^− 3^), Wilcoxon rank-sum test) (Fig. 5). While it is possible that bleeding could contribute to an increase of initial dd-cfDNA levels, our finding suggests a potential link to a delayed stabilization status of grafted organ.

## dd-cfDNA sensitively indicates immunosuppression responses in KTx patients

**Fig. 6:**
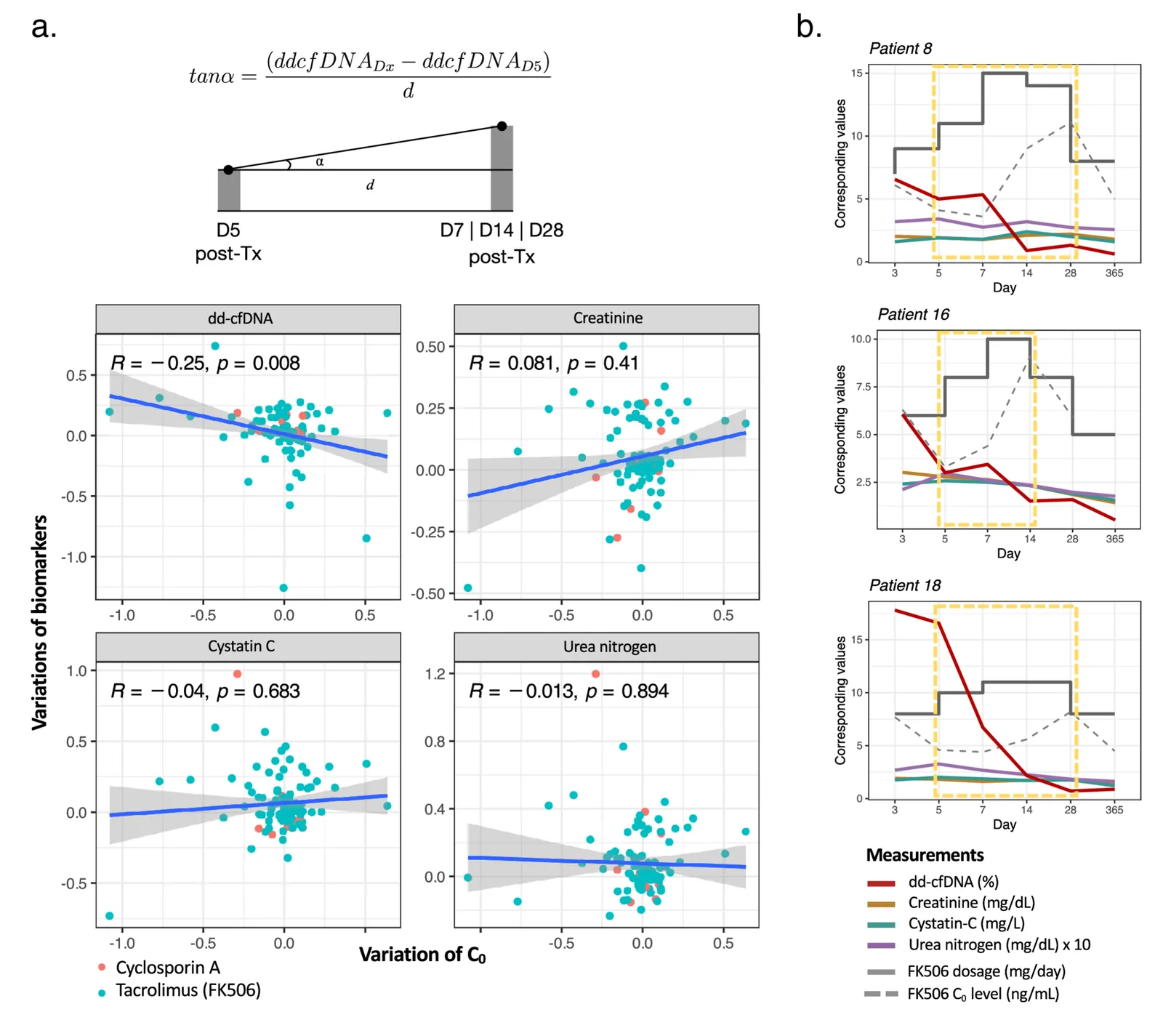
The relationship between clinical serum-based measurements and immunosuppression levels in KTx patients. **a** Demonstration of estimating longitudinal changes by calculating the tangent of the slope formed by the measured values on each subsequent day relative to the baseline day (defined here as day 5 post-Tx) and the temporal distance between measurements. Scatter plots show individual correlations between longitudinal changes of each type of serum-based measurements (including dd-cfDNA) and those of tacrolimus trough level (FK506 C_0_), Spearman’s rho (R) and *p*-value are shown; **b** Representative cases demonstrating sharp decreases in dd-cfDNA levels, which negatively correspond to both consecutive increases in tacrolimus dosage and succeeding increases in C_0_ level (highlighted in dashed yellow boxes)

Among the 39 kidney transplant recipients, nearly 25% required consecutive increases in immunosuppressive therapy (i.e., increased tacrolimus dosage). We evaluated how these adjustments were reflected in dd-cfDNA and conventional serum markers. Day 5 post-transplant (D5) was used as baseline. Longitudinal changes were quantified by calculating the slope tangent between each time point and D5 baseline (Fig. 6a). Tangent values were individually computed for dd-cfDNA, three described serum markers, and tacrolimus trough levels (C_0_) (Supplemental Material 2).

We examined the pairwise correlations and found that longitudinal changes in dd-cfDNA levels were marginally and negatively correlated with that of tacrolimus C_0_ levels (Spearman’s rho = −0.25, *p *= 8 × 10^− 3^) (Fig. 6a). Hence, increased tacrolimus C_0_ was linked to lower dd-cfDNA levels, and vice versa. No such correlation was observed for creatinine, cystatin C, or urea nitrogen. Notably, significant negative correlations between longitudinal changes in dd-cfDNA and tacrolimus trough levels were observed only on days 14 and 28 post-Tx, suggesting that the marker exhibits greater responsiveness once stable graft conditions are established (Fig.S7).

In several representative cases with consecutive increases of tacrolimus dosage (patients #8, #16 and #18), dd-cfDNA showed a clear inverse trend with both dosage and C_0_ levels (Fig. 6b). The fact that this pattern was absent in other serum markers, highlighting dd-cfDNA’s advantage for rapid assessment of treatment response. This result underscores dd-cfDNA advantage over conventional serum markers for monitoring immunosuppressive therapy in KTx patients.

## Discussion

In this study, we applied a population-specific SNP panel and targeted sequencing to 322 samples from 39 KTx recipients. Elevated dd-cfDNA accurately identified graft injury, particularly biopsy-confirmed AR, and matched conventional markers in rejection discrimination. Early post-transplant stabilization of dd-cfDNA was likely influenced by surgical factors such as donor bleeding. Additionally, dd-cfDNA levels aligned with histological grading and were elevated before severe stages, suggesting its utility in identifying subclinical injuries. Importantly, dd-cfDNA also indicated immunosuppressant responses, underscoring its value for monitoring and aiding treatment decisions.

Limitations of our study include the limited number of recruited KTx patients. A small number of biopsy-confirmed AR cases restrains the estimation of the area under the curve (AUC) of an optimal dd-cfDNA threshold. In addition, the limited cohort size precluded evaluation of dd-cfDNA performance across different etiologies of graft injury, such as BK polyomavirus–associated nephropathy (BKPyVAN), although none of the analyzed samples was positive for BK viremia. Furthermore, given the long follow-up period (2016–2021) and the fact that biopsies were evaluated contemporaneously with sample collection, only the Banff classification system current at the time of evaluation was used. As a result, some updated chronic active histological parameters, including ti,* i-IF/TA*, and cg scores, were not assessed in our study. On technical aspect, our quantification of dd-cfDNA relies on knowing both donor and recipient’s genotypes, restraining its use case in cadaveric KTx. Alternative methods using only the recipient’s genotype were described in our study and others [16, 26, 27].

Recent advances in dd-cfDNA analysis for kidney transplantation include the employment of high-throughput sequencing [16, 18, 21, 22, 28]. Here, we established a cost-effective and scalable protocol using pooled capture-hybridization and high-throughput sequencing for dd-cfDNA quantification, previously validated in liver transplantation [26] and various research contexts [29–32]. By incorporating population-specific SNP probes, we optimized dd-cfDNA quantification for KTx of Japanese patients. On the analytical aspect, some established statistical models help to infer informative SNPs and their related allele frequencies without the need to know the donor’s genotype [27], or even both the donor’s and recipient’s genotypes [33]. However, these approaches may not be reliable when dd-cfDNA fractions reach extremely high level [17]. In our study, all cases utilized living donors with accessible genotypes, enabling precise dd-cfDNA quantification. Future directions may include absolute quantification (copies/mL) to improve diagnostic power and integration with gene expression signatures for more comprehensive monitoring [17, 34].

While dd-cfDNA is known for lacking specificity in distinguishing AR from other kidney injuries [35, 36], its high sensitivity makes it valuable for assessing overall allograft integrity. A growing number of studies have documented the potential of dd-cfDNA as a diagnostic biomarker of AR as well as other types of graft injury in KTx, such as acute tubular necrosis, nephrotoxicity or infection [13, 24]. Recently, a meta-analysis scrutinized 11 studies and obtained a summary receiver operating characteristics (SROC) curve with AUC of 0.83 (pooled sensitivity and specificity of 0.75 and 0.78, respectively) for the dd-cfDNA accuracy in rejection diagnosis, with favorable performance in ABMR diagnosis (SROC AUC of 0.85) [36]. The robust capability of dd-cfDNA in detecting active and chronic active ABMR, but not TCMR or chronic non-rejection-related injuries, was also reported [22, 37, 38]. Concordantly, we observed substantial surges in dd-cfDNA levels during ABMR episodes, in contrast with those of TCMR. Furthermore, as showcased in patient #2, an episode of ABMR accompanied by a concurrent elevation of dd-cfDNA, increased anti-HLA class II reactivity, and the presence of DSA suggests that dd-cfDNA measurement may reflect underlying allosensitization and subclinical immunological activity. The temporal concordance of dd-cfDNA surges with these immunological markers highlights its potential utility for early detection of recurrent humoral injury.

The key advantage of dd-cfDNA monitoring over other serum markers is the wide-ranged and responsive fluctuation, owing to its intrinsic origin of cellular damages. Therefore, dd-cfDNA appears superior for monitoring immunosuppressive responses, not only in detecting AR and subclinical abnormalities that may progress to chronic injury, but also in guiding treatment to avoid drug toxicity. Recently, one study demonstrated that dd-cfDNA was successfully used to stratify rejection risk, leading to a reduction of mycophenolic mofetil in low-risk patients [39]. Also, decrease of dd-cfDNA levels following anti-rejection treatments were reported, though observed cases were rather sporadic [15]. Our study is the first to statistically demonstrate that longitudinal changes in dd-cfDNA levels reflect kidney transplant recipients’ responses to immunosuppressant dosing, with a significant negative correlation not observed in conventional serum markers.

In conclusion, we demonstrated the elevated dd-cfDNA levels during diverse forms of acute rejection and graft injury in KTx patients. Our findings support the applicability of dd-cfDNA as a molecular biomarker for monitoring kidney allograft integrity and guiding immunosuppressive treatment. This study also provides proof-of-concept for our customized, cost-effective target-capture sequencing protocol for dd-cfDNA quantification, demonstrating its scalability for large sample sets and patient cohorts.

## Supplementary Information

Below is the link to the electronic supplementary material.

Supplementary Material 1

Supplementary Material 2

Supplementary Material 3

Supplementary Material 4

## Data Availability

The raw sequencing data generated in the current study are available from the corresponding author upon reasonable request. All calculated dd-cfDNA levels, sample characteristics and various clinical parameters are provided in Supplemental Material 1. The analysis data of immunosuppressant longitudinal changes are provided in Supplemental Material 2. The list of 1000 SNPs employed in our target-sequencing protocol is disclosed in Supplemental Material 3.

## References

[1] Tonelli M, Wiebe N, Knoll G, Bello A, Browne S, Jadhav D, et al. Systematic review: kidney transplantation compared with dialysis in clinically relevant outcomes. Am J Transplant. 2011. 10.1111/j.1600-6143.2011.03686.x 10.1111/j.1600-6143.2011.03686.x 21883901

[2] Lentine KL, Smith JM, Lyden GR, Miller JM, Dolan TG, Bradbrook K, et al. Am J Transpl. 2024;24(2S1):S19–118. 2024.01.012 OPTN/SRTR 2022 Annual Data Report: Kidney. 10.1016/j.ajt.2024.01.012 38431360

[3] Marcén R, Fernández-Rodriguez A, Rodríguez-Mendiola N, Ponte B, Galeano C, Villafruela JJ et al. Evolution of rejection rates and kidney graft survival: a historical analysis. Transplant Proc. 2009;41(6):2357–9. 10.1016/j.transproceed.2009.06.049 10.1016/j.transproceed.2009.06.049 19715918

[4] Boratyńska M, Szepietowski T, Szewczyk Z. Acute rejection and delayed graft function–risk factors of graft loss. Annals transplantation: Q Pol Transplantation Soc. 1996;1(2):19–22. 9869925

[5] Roufosse C, Simmonds N, Clahsen-Van Groningen M, Haas M, Henriksen KJ, Horsfield C, et al. A 2018 Reference Guide to the Banff Classification of Renal Allograft Pathology. Transplantation. 2018. 10.1097./TP.0000000000002366. 10.1097/TP.0000000000002366 PMC7597974 30028786

[6] Cooper JE. Evaluation and treatment of acute rejection in kidney allografts. Clin J Am Soc Nephrol. 2020;15(3):430–8. 10.2215/CJN.11991019. 10.2215/CJN.11991019 PMC7057293 32066593

[7] Do H, Lucy R, Wong G, Hon W. The Evolution of HLA-Matching in Kidney Transplantation. In: Current Issues and Future Direction in Kidney Transplantation [Internet]. InTech; 2013 [cited 2020 Aug 28]. Available from: http://www.intechopen.com/books/current-issues-and-future-direction-in-kidney-transplantation/the-evolution-of-hla-matching-in-kidney-transplantation10.5772/54747

[8] Josephson MA. Monitoring and managing graft health in the kidney transplant recipient. Clin J Am Soc Nephrol. 2011. 10.2215/CJN.01230211. 10.2215/CJN.01230211 21734093

[9] Haas M, Loupy A, Lefaucheur C, Roufosse C, Glotz D, Seron D, et al. The Banff 2017 kidney meeting report: revised diagnostic criteria for chronic active T cell–mediated rejection, antibody-mediated rejection, and prospects for integrative endpoints for next‐generation clinical trials. Am J Transplant. 2018;18(2):293–307. 10.1111/ajt.14625. 29243394 10.1111/ajt.14625 PMC5817248

[10] Rush D, Arlen D, Boucher A, Busque S, Cockfield SM, Girardin C, et al. Lack of benefit of early protocol biopsies in renal transplant patients receiving TAC and MMF: a randomized study. Am J Transplant. 2007. 10.1111/j.1600-6143.2007.01979.x. 17908280 10.1111/j.1600-6143.2007.01979.x

[11] Moein M, Papa S, Ortiz N, Saidi R. Protocol biopsy after kidney transplant: clinical application and efficacy to detect allograft rejection. Cureus. 2023. 10.7759/CUREUS.34505 10.7759/cureus.34505 PMC9983784 36874304

[12] Lo YMD, Tein MSC, Pang CCP, Yeung CK, Tong KL, Magnus Hjelm N. Presence of donor-specific DNA in plasma of kidney and liver-transplant recipients. Lancet. 1998;351(9112):1329–30. 10.1016/S0140-6736(. 05)79055-3 PubMed PMID: 9643800. 9643800 10.1016/s0140-6736(05)79055-3

[13] Moreira VG, García BP, Martín JMB, Suárez FO, Alvarez FV. Cell-free DNA as a noninvasive acute rejection marker in renal transplantation. Clin Chem. 2009. 10.1373/clinchem.2009.129072 . PubMed PMID: 19729469. 10.1373/clinchem.2009.129072 19729469

[14] Puliyanda DP, Swinford R, Pizzo H, Garrison J, De Golovine AM, Jordan SC. Donor-derived cell-free DNA (dd-cfDNA) for detection of allograft rejection in pediatric kidney transplants. Pediatr Transpl. 2021;25(2):e13850. 10.1111/PETR.13850. 10.1111/petr.13850 33217125

[15] Benning L, Morath C, Fink A, Rudek M, Speer C, Kälble F, et al. Donor-derived cell-free DNA (dd-cfDNA) in kidney transplant recipients with indication biopsy-results of a prospective single-center trial. Transpl Int. 2023;36. 10.3389/ti.2023.11899. 10.3389/ti.2023.11899 PMC10654198 38020751

[16] Zhang H, Zheng C, Li X, Fu Q, Li J, Su Q, et al. Diagnostic performance of donor-derived plasma cell-free DNA fraction for antibody-mediated rejection in post renal transplant recipients: a prospective observational study. Front Immunol. 2020. 10.3389/fimmu.2020.00342 . PubMed PMID: 32184785. 32184785 10.3389/fimmu.2020.00342 PMC7058974

[17] Oellerich M, Shipkova M, Asendorf T, Walson PD, Schauerte V, Mettenmeyer N, et al. Absolute quantification of donor-derived cell-free DNA as a marker of rejection and graft injury in kidney transplantation: results from a prospective observational study. Am J Transpl. 2019;19(11):3087–99. 10.1111/AJT.15416. 10.1111/ajt.15416 PMC6899936 31062511

[18] Grskovic M, Hiller DJ, Eubank LA, Sninsky JJ, Christopherson C, Collins JP, et al. Validation of a clinical-grade assay to measure donor-derived cell-free DNA in solid organ transplant recipients. J Mol Diagn. 2016. 10.1016/j.jmoldx.2016.07.003 . 27727019 10.1016/j.jmoldx.2016.07.003

[19] Nie W, Su X, Liu L, Li J, Fu Q, Li X, et al. Dynamics of donor-derived cell-free DNA at the early phase after pediatric kidney transplantation: a prospective cohort study. Front Med (Lausanne). 2022;8:814517. 10.3389/FMED.2021.814517/FULL. 35071284 10.3389/fmed.2021.814517 PMC8777035

[20] Jordan SC, Bunnapradist S, Bromberg JS, Langone AJ, Hiller D, Yee JP, et al. Donor-derived cell-free DNA identifies antibody-mediated rejection in donor specific antibody positive kidney transplant recipients. Transpl Direct. 2018. 10.1097/TXD.0000000000000821. 10.1097/TXD.0000000000000821 PMC6133406 30234148

[21] Gielis EM, Ledeganck KJ, Dendooven A, Meysman P, Beirnaert C, Laukens K, et al. The use of plasma donor-derived, cell-free DNA to monitor acute rejection after kidney transplantation. Nephrol Dialysis Transplantation. 2020. 10.1093/ndt/gfz091 . PubMed PMID: 31106364. 10.1093/ndt/gfz091 31106364

[22] Bloom RD, Bromberg JS, Poggio ED, Bunnapradist S, Langone AJ, Sood P, et al. Cell-free DNA and active rejection in kidney allografts. J Am Soc Nephrol. 2017;28(7):2221–32. 10.1681/ASN.2016091034 28280140 10.1681/ASN.2016091034 PMC5491290

[23] Gielis EM, Ledeganck KJ, De Winter BY, Del Favero J, Bosmans JL, Claas FHJ, et al. Cell-free DNA: an upcoming biomarker in transplantation. Am J Transplant. 2015. 10.1111/ajt.13387 . PubMed PMID: 26184824. 26184824 10.1111/ajt.13387

[24] Knight SR, Thorne A, Lo Faro ML. Donor-specific cell-free DNA as a biomarker in solid organ transplantation. Syst Rev Transp. 2019. 10.1097/TP.0000000000002482 10.1097/TP.0000000000002482 30308576

[25] Martuszewski A, Paluszkiewicz P, Król M, Banasik M, Kepinska M. Donor-derived cell-free DNA in kidney transplantation as a potential rejection biomarker: a systematic literature review. J Clin Med 2021. 2021;10(2):193. 10.3390/JCM10020193. 10.3390/jcm10020193 PMC7827757 33430458

[26] Kanamori H, Yamada Y, Ito Y, Shirosaki K, Yamagishi S, Maeda Y, et al. Noninvasive graft monitoring using donor-derived cell-free DNA in Japanese liver transplantation. Hepatol Res. 2024;54(3):300–14. 10. 1111/HEPR.13978 PubMed PMID: 37850337. 37850337 10.1111/hepr.13978

[27] Sharon E, Shi H, Kharbanda S, Koh W, Martin LR, Khush KK, et al. Quantification of transplant-derived circulating cell-free DNA in absence of a donor genotype. PLoS Comput Biol. 2017. 10.1371/journal.pcbi.1005629 . 28771616 10.1371/journal.pcbi.1005629 PMC5542400

[28] Sigdel T, Archila F, Constantin T, Prins S, Liberto J, Damm I, et al. Optimizing detection of kidney transplant injury by assessment of donor-derived cell-free DNA via massively multiplex PCR. J Clin Med. 2018;8(1):19. 10.3390/jcm8010019. 30583588 10.3390/jcm8010019 PMC6352163

[29] Nakaoka H, Gurumurthy A, Hayano T, Ahmadloo S, Omer WH, Yoshihara K, et al. Allelic imbalance in regulation of ANRIL through chromatin interaction at 9p21 endometriosis risk locus. PLoS Genet. 2016;12(4). 10.1371/journal.pgen.1005893. 10.1371/journal.pgen.1005893 PMC4824487 27055116

[30] Yamaguchi M, Nakaoka H, Suda K, Yoshihara K, Ishiguro T, Yachida N, et al. Spatiotemporal dynamics of clonal selection and diversification in normal endometrial epithelium. Nat Commun 2022. 2022;13(1):1. 10.1038/s41467-022-28568-2 . 10.1038/s41467-022-28568-2 PMC8854701 35177608

[31] Jinam TA, Hosomichi K, Nakaoka H, Phipps ME, Saitou N, Inoue I. Allelic and haplotypic HLA diversity in indigenous Malaysian populations explored using next generation sequencing. Hum Immunol. 2022;83(1):17–26. 10.1016/J.HUMIMM.2021.09.005 10.1016/j.humimm.2021.09.005 34615609

[32] Ahmadloo S, Nakaoka H, Hayano T, Hosomichi K, You H, Utsuno E, et al. Rapid and cost-effective high-throughput sequencing for identification of germline mutations of BRCA1 and BRCA2. J Hum Genet. 2017;62(5):561–7. 10.1038/jhg.2017.5. 28179634 10.1038/jhg.2017.5

[33] Gordon PMK, Khan A, Sajid U, Chang N, Suresh V, Dimnik L, et al. An algorithm measuring donor cell-free DNA in plasma of cellular and solid organ transplant recipients that does not require donor or recipient genotyping. Front Cardiovasc Med. 2016. 10.3389/fcvm.2016.00033. 27713880 10.3389/fcvm.2016.00033 PMC5031701

[34] Halloran PF, Reeve J, Madill-Thomsen KS, Kaur N, Ahmed E, Cantos C, et al. Combining donor-derived cell-free DNA fraction and quantity to detect kidney transplant rejection using molecular diagnoses and histology as confirmation. Transplantation. 2022;106(12):2435–42. 10.1097/TP.0000000000004212 10.1097/TP.0000000000004212 PMC9698190 35765145

[35] Pagliazzi A, Bestard O, Naesens M, Donor-derived cell-free DNA. attractive biomarker seeks a context of use. Transpl Int. 2023;36. 10.3389/.TI.2023.12406. 10.3389/ti.2023.12406 PMC10722252 38106814

[36] Yang H, Wang D, Sun X, Wang H, Lan Y, Wei L. Diagnostic performance of GcfDNA in kidney allograft rejection: a meta-analysis. Front Physiol. 2024;14. 10.3389/fphys.2023.1293402. 10.3389/fphys.2023.1293402 PMC10803602 38264334

[37] Huang E, Sethi S, Peng A, Najjar R, Mirocha J, Haas M, et al. Early clinical experience using donor-derived cell-free DNA to detect rejection in kidney transplant recipients. Am J Transplant. 2019;19(6):1663–70. 10.1111/ajt.15289. 30725531 10.1111/ajt.15289

[38] Aubert O, Ursule-Dufait C, Brousse R, Gueguen J, Racapé M, Raynaud M, et al. Cell-free DNA for the detection of kidney allograft rejection. Nat Med. 2024;30(8):2320–7. 10.1038/S41591-024-03087-3. 10.1038/s41591-024-03087-3 PMC11333280 38824959

[39] Osuchukwu G, Trevino A, McCormick S, Kaur N, Prigmore B, Al Haj Baddar N, et al. Use of Donor-derived cell-free DNA to inform tapering of immunosuppression therapy in kidney transplant recipients: an observational study. Transpl Direct. 2024;10(4):E1610. 10.1097/TXD.0000000000001610 10.1097/TXD.0000000000001610 PMC10936987 38481964

