# Supplementary material for: Applications of donor-derived cell-free DNA in kidney transplantation healthcare: view from a prospective single-center study
Source: Clin Exp Nephrol. 2026 Jun 2;30(9):1368–79. doi: 10.1007/s10157-026-02889-8 (PMC13518428; doi:10.1007/s10157-026-02889-8)
Supplement: Supplementary file 1 [file 10157_2026_2889_MOESM4_ESM.docx]

**SUPPLEMENTARY INFORMATION**

1. **SUPPLEMENTARY METHODS**

**Designing of SNPs targeting probes**

We selected 1000 SNPs across all autosomes of which minor allele frequency (MAF) is between 0.4 and 0.5 based on the reported frequencies in Japanese population [1] (Supplemental Data 2). Assuming Hardy–Weinberg equilibrium, desired MAF value is expected to be of 23 to 25% homozygous in both donor and recipient, and the theoretical probability of both donor and recipient having a different allele is 11.5 to 12.5% [2]. Probes for capture-hybridization of cell-free DNA (cfDNA) targeting each of 1000 SNPs were purchased from Roche as SeqCap EZ Library (Nippon Genetics, Japan). In new batch of long-term samples, we used a refined version of probes (including 300 SNPs shortlisted from the original 1000 SNP panel) based on 3.5KJPNv2 database [3], provided by SeqCap EZ Prime Choice (Roche, Switzerland).

**Cell-free DNA extraction, probe-capture protocol and sequencing procedure**

Blood samples were collected in 10ml PAXgene® Blood ccfDNA tube (QIAGEN, Germany) by peripheral venesection and plasma was separated according to manufacturer’s instruction. Plasma was stored at -80^o^C until cfDNA extraction. Cell-free DNA was isolated from 1 mL of the recipient’s plasma using the QIAamp MinElute ccfDNA Mini Kit (QIAGEN, Germany). The cfDNA was eluted with 33 µL of ultra-pure water and 24 µL of it was used for DNA repair with NEBNext FFPE DNA Repair Mix (New England Biolabs, USA). Libraries were prepared using the repaired cfDNA and NEBNext Ultra II DNA Library Prep Kit. After minimal amplification by PCR, target SNPs were enriched by capture-hybridization using KAPA HyperCap Target Enrichment Probes and KAPA HyperCapture Reagent Kit (Roche, Switzerland). Capture-hybridization was performed using our designed probe sets, following our single-reaction protocol which was previously described [4]. The enriched library was amplified by 18 cycles of PCR and sequenced using the MiSeq or NovaSeq 6000 platform (Illumina, USA). Where kits were used, all manufacturer's instructions were followed.

**Genotyping of target SNPs from donors and recipients**

Raw *fastq* reads obtained from sequencing facility were trimmed of sequencing adaptors and performed quality control using Trimmomatic-0.38 and the following parameters: leading:15 / trailing:15 / slidingwindow:4:20 / crop:220 / minlen:36. Trimmed sequences were mapped to reference human genome hg19 using bwa-mem followed by quality controls using picard/MarkDuplicates and samtools v1.18. Variant call was conducted using HaplotypeCaller tool of GATK v4.2.5.0 package [5] using options: –output-mode EMIT_ALL_ACTIVE_SITES and –emit-ref-confidence BP_RESOLUTION. Then filtering steps on resulted vcf files were performed using GATK/VariantFiltration tool with parameters: FS < 60.00 / QD > 2 / MQ > 40 / DP ≥ 30. Heterozygous sites in recipients were excluded before selection of informative SNPs.

**Quantification of donor-derived cell-free DNA**

After genotyping of 1000 SNPs, we selected informative SNPs personalized for each donor-recipient pair that satisfy either one in two conditions: (1) homozygous genotype is different between donor and recipient, or (2) genotype of recipient is homozygous and that of donor is heterozygous. The counting the reads mapped on each informative SNP was performed by GATK/DepthOfCoverage tool. Among pairs of donor-recipient, we identified 165 to nearly 400 informative SNPs out of a total of 1000 candidate SNPs. Parent-child and sibling pairs are expected to share common allele, hence, typically have fewer informative SNPs (Figure S1). For dd-cfDNA quantification, only samples with at least 50 eligible informative SNPs are considered. Informative SNP locus must satisfy coverage depth ≥ 5, minimum mapping quality ≥ 30 and base quality ≥ 26. Subsequently, summarized ratios of donor-derived read counts on the total mapped reads were calculated by in-house scripts, and resulted dd-cfDNA fraction was represented in percentage.

**Statistical analyses**

Statistical analyses were performed using *Hmisc* package in R-software. The Wilcoxon rank-sum test and Type III repeated measures ANOVA were used for comparisons between independent groups and for continuous dependent variables, respectively. Spearman’s correlation was used to evaluate relationship between residues. A p-value less than 0.05 is considered to be statistically significant. Visualization of the data was done using the *ggplot2* package in R.

**REFERENCES**

1.	Higasa K, Miyake N, Yoshimura J, et al (2016) Human genetic variation database, a reference database of genetic variations in the Japanese population. Journal of Human Genetics 2016 61:6 61:547–553

2.	Beck J, Bierau S, Balzer S, et al (2013) Digital droplet PCR for rapid quantification of donor DNA in the circulation of transplant recipients as a potential universal biomarker of graft injury. Clin Chem 59:1732–1741

3.	Tadaka S, Katsuoka F, Ueki M, et al (2019) 3.5KJPNv2: an allele frequency panel of 3552 Japanese individuals including the X chromosome. Human Genome Variation 2019 6:1 6:1–9

4.	Ahmadloo S, Nakaoka H, Hayano T, et al (2017) Rapid and cost-effective high-throughput sequencing for identification of germline mutations of BRCA1 and BRCA2. J Hum Genet 62:561–567

5.	Poplin R, Ruano-Rubio V, DePristo MA, et al (2018) Scaling accurate genetic variant discovery to tens of thousands of samples. bioRxiv 201178

2. **SUPPLEMENTARY FIGURES**

**Fig.S1** Number of informative SNPs detected between each pair of donor and recipient, via genotyping of 1000 SNP loci (donor-recipient pair #14 opted out from current study). Informative SNPs were stratified based on the donor's genotype: termed as donor heterozygote SNP if the genotype of donor is heterozygous and that of recipient is homozygous, and donor homozygote SNP if homozygous genotype is different between donor and recipient

**Fig.S2** Detail demonstrations of measurements from all patients with biopsy-confirmed rejections (n=6), including (A) four longitudinal cases and (B) two cross-sectional cases; (C) two cases with severe kidney injuries not related to rejection are also presented. Abbreviations: aT.BIA = acute T cell–mediated rejection Banff IA; aT.BIIA = acute T cell–mediated rejection Banff IIA; aT.BIB = acute T cell–mediated rejection Banff IB; aT.BIIB = acute T cell–mediated rejection Banff IIB; aA. = acute antibody–mediated rejection

**Fig.S3** Comparison of levels of dd-cfDNA (%), creatinine (mg/dL), cystatin C (mg/dL), and blood urea nitrogen (mg/dL) between the group of samples with biopsy-confirmed rejection episodes and the group of samples without histologically detectable rejection

**Type III Repeated measures ANOVA test**

F = 1.198

p-value = 0.342

**Fig.S4** Examining the effect of biopsy procedure on the fluctuation of dd-cfDNA levels. Red lines connect dd-cfDNA levels of the same patient at the day of biopsy (Bx), 1 day after biopsy (Bx+1) and 2 days after biopsy (Bx+2). Type III Repeated measures ANOVA test was performed

**Fig.S5** Pairwise correlations between each of the three serum markers (i.e., creatinine, cystatin C and urea nitrogen) and dd-cfDNA level among different groups of KTx patient. (A) Correlations in the group without suspected rejection (‘eventless’ group) (Right panel derived from Figure 2A and 2B); (B) Correlations in the group with suspected rejection episodes (‘event’ group) (Right panel derived from Figure 2C and 2D). Spearman correlation tests were performed

**Fig.S6** Distribution of dd-cfDNA levels across various histological assessment of kidney integrity. (A) Distribution of dd-cfDNA levels across various Banff Lesion Score categories, with the severity of the condition increases as the score moves to the right. Abbreviation of Banff Lesion Score categories: *i* = Interstitial inflammation, *t* = Tubulitis, *g* = Glomerulitis, *v* = Intimal arteritis, *ci* = Interstitial fibrosis, *ct* = Tubular atrophy, *ptc* = Peritubular capillaritis, *C4d* = C4d staining scores, *ptcbm* = peritubular capillary basement membrane, *ah* = Arteriolar hyalinosis, *aah* = Hyaline arteriolar thickening; (B) Distribution of dd-cfDNA levels across different grades of chronic renal injury assessments, including IF/TA and Arteriosclerosis. Grading annotations: MILD=mild; SEMIMOD=semi-moderate; MOD=moderate; SEMISEVERE=semi-severe; SEVERE=severe

**Fig.S7** Scatter plots show individual correlations between longitudinal change values from each serum-based measurement (including dd-cfDNA) and the corresponding values calculated from FK506 C_0_. These correlations were facetted by timepoints compared to the baseline day (Day 5), represented by three columns corresponding to Day 7, Day 14 and Day 28 post-Tx
